# Editorial: Astrocytes and their crucial role in modulating neurotransmission

**DOI:** 10.3389/fncel.2025.1749299

**Published:** 2025-12-02

**Authors:** Sonia Luz Albarracin, Fabiola M. Ribeiro

**Affiliations:** 1 Departamento de Nutrición y Bioquímica, Pontificia Universidad Javeriana, Bogota, Colombia; 2 Department of Biochemistry and Immunology, Universidade Federal de Minas Gerais, Belo Horizonte, Brazil

**Keywords:** astrocyte, neurotransmission, neuroinflammation, plasticity, neuronal synchrony, calcium

Astrocytes represent the most abundant glial cell population in the brain, where they fulfill critical physiological roles and are implicated in a wide spectrum of neurological diseases (Lee et al., 2022; de Lima and Ribeiro, 2023). Traditionally considered support cells, astrocytes are now recognized as key players in shaping neurotransmission and neuronal communication. Moreover, these glial cells play an important role in synaptogenesis and in the phagocytosis of dead cells, dystrophic synapses, and myelin (Trachtenberg et al., 2002; Kucukdereli et al., 2011; Paolicelli et al., 2011). However, in disease states, these cells can become reactive and release inflammatory factors, which may affect outcomes for better or worse (Moulson et al., 2021; Verkhratsky et al., 2021). The Research Topic entitled “*Astrocytes and their crucial role in modulating neurotransmission*,” which includes three reviews, one method and one original research paper, highlights the most up-to-date information about these fascinating glial cells. These articles comprehensively discuss the role of astrocytes in neuroinflammation, synaptic plasticity, and neurotransmission. By synthesizing the most current research, these reports aim to deepen our understanding of astrocytic functions and solidify their role as crucial modulators of neurotransmission.

The review article by Ammothumkandy et al. provides a critical framework for understanding the role of astrocytes in one of the most fundamental patterns of brain activity: neuronal synchrony. This work compellingly argues that astrocytes are central conductors of neural ensemble activity, moving beyond their role at single synapses to modulate entire networks (Buskila et al., 2019; Doron et al., 2022). The authors also discuss how astroglia transition from a normal to a pathological state, favoring epileptogenic seizures and cognitive decline (Lee et al., 2014; Diaz Verdugo et al., 2019). This review is paramount to our topic as it elegantly connects the molecular mechanisms of astrocytic modulation to the emergent functional (or pathological) outcomes of synchronized rhythms, seen in everything from memory consolidation to epileptic seizures. It firmly establishes that to understand the brain's large-scale communicative patterns, we must look beyond neurons and include astroglia as key players in synchronized spontaneous neuronal activity.

Calcium is a central second messenger in astrocytes. Although there are several software packages to analyze calcium oscillations in brain cells, most of them are not suitable for astrocytes, as these cells exhibit calcium fluctuations that are spatially and temporally different from those observed in neurons (Bazargani and Attwell, 2016; Denizot et al., 2019). The article by Reising et al. describes a machine-learning-driven toolkit, astroCaST, dedicated to analyzing cytosolic calcium oscillations in astrocytes. This software offers comprehensive options for analyzing and interpreting activity events, repurposing existing tools from various data science domains for astrocyte analysis. astroCaST is tailored for astrocytes by being agnostic to the spatial location of events and is capable of handling larger datasets, analyzing more events than previously possible in astrocyte-specific studies. Thus, this software might help us to better understand astrocyte activation and its role in neurotransmission.

Spike timing-dependent plasticity (STDP) is a bidirectional form of synaptic plasticity discovered about 30 years ago and based on the relative timing of pre- and post-synaptic spiking activity with a millisecond precision, which underlies spatial and episodic memory (Debanne and Inglebert, 2023). Astrocytes can modulate STDP by regulating local ion homeostasis and releasing gliotransmitters, such as glutamate and adenosine. Bidirectional communication with neurons is an active modulator of network excitability, synaptic transmission, and plasticity through multiple mechanisms. In their review, Sanz-Gálvez et al. describe how the action potentials can trigger astrocytic release of gliotransmitters that modulate neuronal excitability at the axon initial segment and the conduction velocity at the nodes of Ranvier. Of particular interest are the mechanisms operating in the perinodal region, where astrocytes, via thrombin inhibitors, regulate the myelin–axon junction at the NF155–Caspr1 complex, thereby preventing nodal-space expansion that would otherwise displace sodium channels and slow conduction. Axonal activity is further shown to elicit astrocytic calcium signals mediated by ATP and glutamate, which in turn enhance the release of these same messengers and permit signal propagation across astrocytic networks coupled by connexin 30 (Cx30) and connexin 43 (Cx43) gap junctions. Another mechanism contributing to the fine-tuning of neuronal firing patterns is the release of the S100β protein, which regulates extracellular calcium concentration (Ryczko et al., 2021). Particularly noteworthy is the ability of astrocytes to spontaneously release glutamate, generating slow inward currents in neighboring neurons, together with the actions of other gliotransmitters—such as D-serine, ATP, adenosine, and S100β–that exert a substantial influence on the regulation of synaptic plasticity.

This critical regulatory role also makes astrocytes vulnerable targets in pathology, exemplified by their central involvement in Major Depressive Disorder (MDD) and ischemic stroke. In their review, Puentes-Orozco et al. discuss how chronic stress in MDD triggers persistent neuroinflammation and astrocytic dysfunction. The crucial factor appears to be the balance between astrocytic phenotypes; the neurotoxic A1 phenotype, which exacerbates inflammation and impairs neuroplasticity, leading to dendritic atrophy in the hippocampus. In contrast with the neurotrophic A2 phenotype, which promotes neuronal survival and repair (Fan and Huo, 2021). Antidepressants, including selective serotonin reuptake inhibitors (SSRIs), may exert their therapeutic effects not just by targeting monoamines, but by reducing pro-inflammatory cytokines and potentially encouraging the beneficial A2 phenotype transition.

In the context of acute injury, such as ischemic stroke, the resulting neuronal damage and inflammation also lead to an increased population of reactive astrocytes (Knight-Greenfield et al., 2019), and astrocyte reprogramming (Peng et al., 2022). In their work, Clark et al. shift the focus toward directly exploiting the proliferative capacity of these glial cells for repair. Using a canine stroke model, they demonstrated that delivery of a viral vector (AAV) encoding the transcription factor NeuroD1 could reprogram astrocytes into neurons. This treatment was associated with faster and greater behavioral recovery, reduced ventricular enlargement, suggesting less tissue atrophy, and, notably, decreased levels of astrocyte and microglial activation in treated animals compared to controls. These findings raise the possibility that converting reactive astrocytes into neurons may mitigate the inflammatory response, as the reprogrammed cells no longer produce pro-inflammatory cytokines like IL-6 and TNF-α.

Taken together, the contributions in this Research Topics provide a comprehensive and timely overview of the crucial role of astrocytes in modulating neurotransmission and their implications in neurological diseases. Historically seen as mere support cells, astrocytes now emerge as essential actors in neuronal communication, synaptogenesis, and the development of pathological states like epilepsy and Major Depressive Disorder (MDD). Specific articles within the topic discuss how astrocytes act as central conductors of neuronal synchrony and examine the intricate mechanisms by which they modulate synaptic strength, such as Spike Timing-Dependent Plasticity (STDP), through the release of gliotransmitters. Furthermore, the Research Topic presents a machine-learning toolkit (astroCaST) for accurately analyzing astrocytic calcium signaling and explores therapeutic approaches, such as reprogramming reactive astrocytes into neurons following ischemic stroke.
